# Selection of Filovirus Isolates for Vaccine Development Programs

**DOI:** 10.3390/vaccines9091045

**Published:** 2021-09-19

**Authors:** Daniel N. Wolfe, Carol L. Sabourin, Michael J. Merchlinsky, William C. Florence, Larry A. Wolfraim, Kimberly L. Taylor, Lucy A. Ward

**Affiliations:** 1 U.S. Department of Health and Human Services (DHHS), Assistant Secretary for Preparedness and Response (ASPR), Biomedical Advanced Research and Development Authority (BARDA), Washington, DC 20201, USA; Michael.merchlinsky@hhs.gov; 2 Tunnell Government Services, Inc., Supporting Biomedical Advanced Research & Development Authority (BARDA), Assistant Secretary for Preparedness and Response (ASPR), U.S. Department of Health and Human Services (DHHS), Washington, DC 20201, USA; carol.sabourin@hhs.gov; 3 U.S. Department of Health and Human Services (DHHS), National Institutes of Health (NIH), National Institute of Allergy and Infectious Diseases (NIAID), Rockville, MD 20852, USA; clint.florence@nih.gov (W.C.F.); larry.wolfraim@nih.gov (L.A.W.); Kimberly.taylor3@nih.gov (K.L.T.); 4 U.S. Department of Defense (DOD), Joint Program Executive Office for Chemical, Biological, Radiological and Nuclear Defense (JPEO-CBRND), Joint Project Manager for Chemical, Biological, Radiological, and Nuclear Medical (JPM CBRN Medical), Fort Detrick, MD 21702, USA; lucy.a.ward.civ@mail.mil

**Keywords:** ebola, Sudan, Marburg, filovirus, animal rule, viral isolate, challenge strain

## Abstract

The continuing outbreaks of ebola virus disease highlight the ongoing threat posed by filoviruses. Fortunately, licensed vaccines and therapeutics are now available for *Zaire ebolavirus*. However, effective medical countermeasures, such as vaccines for other filoviruses such as *Sudan ebolavirus* and the Marburg virus, are presently in early stages of development and, in the absence of a large outbreak, would require regulatory approval via the U.S. Food and Drug Administration (FDA) Animal Rule. The selection of an appropriate animal model and virus challenge isolates for nonclinical studies are critical aspects of the development program. Here, we have focused on the recommendation of challenge isolates for *Sudan ebolavirus* and Marburg virus. Based on analyses led by the Filovirus Animal and Nonclinical Group (FANG) and considerations for strain selection under the FDA Guidance for the Animal Rule, we propose prototype virus isolates for use in nonclinical challenge studies.

## 1. Introduction

The *Filoviridae* family represents a group of filamentous, single-stranded, negative-sense RNA viruses known as the filoviruses [1,2]. Only viruses belonging to the Marburgvirus and Ebolavirus genera can cause severe disease in humans and nonhuman primates. The first documented filovirus infections were contracted from exposure to nonhuman primates carrying Marburg virus (MARV), in 1967 [1]. Ebolaviruses were first discovered in 1976, with at least six known distinct species now identified, including ebola virus or EBOV (species *Zaire ebolavirus*); Sudan virus or SUDV (species *Sudan ebolavirus*); Taï Forest virus or TAFV (species *Taï Forest ebolavirus*, formerly Côte d’Ivoire ebolavirus); Bundibugyo virus or BDBV (species *Bundibugyo ebolavirus*), Reston virus (species *Reston ebolavirus*), and Bombali virus (species *Bombali ebolavirus*) [1,2]. Of these five, four (EBOV, SUDV, TAFV, BDBV) have virus isolates obtained from infected humans [2].

The largest outbreak of ebolavirus disease (EVD) occurred in 2014–2016, resulting in more than 28,000 cases and 11,000 deaths as a result of infections by the EBOV, which triggered global investments to advance the development of medical countermeasures. For the vaccine field, this ultimately resulted in the licensure of a vesicular stomatitis virus-vectored chimera expressing the EBOV glycoprotein, ERVEBO^®^, by the FDA, as well as the authorization of Zabdeno^®^ and Mvabea^®^ as a two-dose vaccine by the European Medicines Agency. Importantly, countermeasures were available for testing by the end of the West Africa outbreak and the more recent EVD outbreaks from 2018 to 2020, providing additional clinical data for vaccine efficacy [3,4]. The zoonotic nature of these viruses and their encroachment into areas of viral reservoirs will continue to drive sporadic outbreaks of EVD and Marburg virus disease (MVD). We can expect the viruses to continue to evolve in their natural hosts, resulting in new strains, some of which may be infectious in humans. This is exemplified by the observation of novel strains of filoviruses circulating in bats when surveillance is extended into wild animals. However, there are currently no licensed vaccines for filoviruses other than EBOV, and most candidates are in the preclinical stages of development.

The SUDV has caused seven outbreaks of SUDV disease (SDV) to date, with the most consequential occurring in South Sudan in 2000, infecting over 400 people with a case fatality ratio of 53 percent [5]. The combined case fatality ratio for all of the documented infections by SUDV has been greater than 50 percent. The most common clinical symptom observed is fever, found in 85 percent of cases. Fever was often accompanied by headaches, muscle aches, and weakness. The incubation period ranged from two to 21 days, and death resulted from dehydration and organ failure, typically within 10 days of symptom onset. Subsequent analyses of peripheral blood mononuclear cell samples showed that a lethal outcome following disease correlated with higher levels of viral RNA [6]. Presently, the only treatment for SDV is palliative care. Of the remaining two ebolavirus species that infect humans, only BDBV has caused sporadic outbreaks, with 206 total confirmed cases between two outbreaks [7], but this will not be a focus of this manuscript.

Marburgviruses are closely related to ebolaviruses in the *Filoviridae* family. A case of MVD was identified in West Africa in August of 2021 and the potential outbreak is still being monitored closely as of September 2021. However, through 2020, twelve different outbreaks of MVD have occurred, with a total of 466 documented cases [8], with the most consequential occurring in 2004–2005 in Angola, resulting in 252 infections and a case fatality ratio of 90 percent. The 2015 review by Glaze et al. provides the most comprehensive review of human clinical data [9]. Clinical data were largely derived from 35 patients diagnosed with MVD. Clinical symptoms generally appeared within three to nine days post-exposure and progressively worsened to include diarrhea, nausea, vomiting, fever, and exanthema or enanthema. The condition of patients usually worsened during the second week of the disease, with some cases resulting in hemorrhaging. Mortality ranged from 20–30 percent in individuals receiving intensive medical care, but reached as high as 80–90 percent in rural settings. The only medical intervention for MVD available is palliative care.

## 2. Role of Vaccines and Development Pathway

The sporadic and unpredictable nature of filovirus outbreaks requires vaccine development plans based on the FDA Animal Rule pathway. The FDA issued the Animal Rule in May 2002, with the title “New Drugs and Biological Products; Evidence Needed to Demonstrate Effectiveness of New Drugs When Human Efficacy Studies Are Not Ethical or Feasible”. The precepts of the Animal Rule are codified in 21 CFR 314.600 through 314.650 for drugs, and 21 CFR 601.90 through 601.95 for biological products, and updated guidance was provided in 2015 [10]. The aim of the Animal Rule is to provide a process for the regulatory evaluation of medical countermeasures when the absence of the disease in nature and the ethics of challenge studies in humans are indefensible, requiring the demonstration of efficacy without a traditional phase III efficacy trial. The initial efforts to evaluate the medical countermeasures against EBOV, including ERVEBO^®^, were carried out using the principles of the Animal Rule. Efficacy was demonstrated in both small animal models and in nonhuman primates infected with a lethal dose of EBOV. Because of these efforts, by the end of the outbreak in 2014, a nominal dose that was demonstrated as safe in phase I trials was available for deployment in a ring vaccination strategy [11].

The deployment of ERVEBO^®^ in the EBOV outbreaks subsequent to 2015 has highlighted the critical role vaccines play as a part of the overall response to filovirus outbreaks. Since approval, more than 300,000 doses of vaccine have been used as part of recent responses to EBOV outbreaks. This has included the vaccination of healthcare workers, front-line responders, and contacts, plus contacts of contacts of index cases. The World Health Organization estimated its efficacy to be 100 percent (95 percent confidence interval 74.7–100 percent) [3], and subsequent analyses of data from vaccine campaigns under expanded access protocols estimated its efficacy to be 97 percent [4].

That said, EBOV vaccines were still in preclinical development by early 2014, with the exception of two earlier clinical trials assessing adenovirus serotype 5 [12] and DNA-based constructs [13]. The West Africa epidemic required an emergency response to expedite vaccine development, resulting in several candidates being pursued, and clinical development being initiated on viral vectored vaccines (adenovirus serotypes 5 and 26, Modified Vaccinia Ankara, chimpanzee adenovirus serotype 3, and vesicular stomatitis virus), DNA constructs, and subunit/virus-like particle candidates [14]. The collective global investments enabled the rapid progress of viral-vectored vaccines into phase II and III clinical trials by early 2015. As of 2021, the pipeline of MARV and SUDV vaccines with clinical data remains limited [15,16]. It is critical that vaccines against MARV and SUDV are pushed into clinical development as soon as feasible to ensure the availability of vaccines for real-world evaluations and use as potential response tools in the event of future outbreaks caused by either of these viruses.

In the absence of a large outbreak in which clinical efficacy would be demonstrated, the current assumption is that U.S. licensure of these products would occur via the FDA Animal Rule pathway [10]. Under this regulatory framework, post-marketing commitments will include plans to demonstrate clinical efficacy in the field, should such an opportunity arise, but licensure would be based on clinical immunogenicity data bridged to nonclinical immunogenicity and efficacy studies, as described for the EBOV vaccine using an adenovirus serotype 26 prime and Modified Vaccinia Ankara boost [17]. However, EBOV also provided a test case for filoviruses, in which the clinical efficacy results could be correlated closely with nonclinical efficacy in nonhuman primate models. Ring vaccinations suggested an onset of protection within ten days [3]. Nonclinical studies in nonhuman primates demonstrated rapid onset to protection as well, with protection against a 1000-pfu intramuscular challenge within seven days post-vaccination [18]. The clinical efficacy demonstrated for EBOV in humans was based on an epidemic of the EBOV Makona strain and utilized a vaccine construct based on a glycoprotein (GP) sequence from a different EBOV strain from the 1995 Kikwit outbreak [11]. Fortunately, the EBOV Makona glycoprotein sequence was sufficiently similar to the EBOV Kikwit GP sequence and no Makona-specific GP constructs were needed. However, for future SUDV and MARV vaccine efforts, this raises the key question of which virus strain(s) to use when conducting important nonclinical efficacy studies and developing key immunological assays.

## 3. Animal Rule Considerations for Isolate Selection

The ability of nonclinical testing to provide predictive value for the effectiveness of vaccines relies on several criteria, which are provided in regulatory guidance from the FDA. It is important to appreciate that, under the Animal Rule, animal models are essentially being utilized as a surrogate for humans since clinical trials are not feasible or ethical. These criteria are inherently tied to whether or not the animal species in question is permissive to the challenge agent, and its subsequent pathophysiological and immunological responses are similar to the human responses. The first factor in choosing a challenge isolate is understanding the clinical history of the isolate in terms of the severity of the disease symptoms caused and whether or not the outcome was lethal. In addition to the clinical history of the virus isolate, there are important scientific considerations to ensure the fidelity of the challenge virus over the lifetime of challenge studies, such as limiting the passage history for RNA viruses to minimize genetic drift, the absence of contaminants, and storage under appropriate conditions to preserve potency. Maintaining the viral isolate in a manner avoiding serial passaging is critical to ensuring that the pathophysiological responses in the animal model will be similar to the human disease. The goal is to identify an isolate that (1) mimics the disease course observed in humans with similar measurable endpoints, (2) is relevant to potential isolates that may be observed, and thus (3) will be of use in predicting the efficacy of vaccines in nonclinical challenge models.

## 4. Technical Considerations

Although the selection of a viral isolate with the biological properties described above is critical for developing an animal model which can be predictive of clinical benefit, there are technical considerations with regard to the production and storage of the challenge virus that are essential to maintain the applicability of the isolate. The filoviruses are single-stranded RNA viruses, and do not exist in nature as a unique nucleotide sequence, but as a distribution of RNA sequences around a consensus sequence that will change with environmental pressure. Although these viruses primarily circulate in wild mammals in nature, their zoonotic nature arises from the fact that the sequence mixture contains some that are compatible with infecting humans. Therefore, we would expect the initial isolates of an outbreak to be highly virulent, with a genetic drift to lower virulence after coadaptation from multiple passage in humans. The consensus viral sequence for EBOV evolved with extended human passage during the outbreak in 2014 [19], and specific changes in the consensus sequence are detected after passage in tissue culture [20]. Therefore, it is imperative to limit the serial passage of the viral isolate in tissue culture in order to prevent the evolution of the virus into a less virulent form. Appropriate quality assurance and quality control measures should be in place to create master and working virus stocks that are characterized in terms of potency (plaque-forming units or pfu/mL and the particle:pfu ratio), identity (genomic sequence to include deep sequencing, Western blots, etc.), and purity (endotoxin, the absence of adventitious agents, etc.), and which are documented with certificates of analysis. When possible, standardized growth protocols using master or working virus stocks will help to minimize variability across laboratories.

Beyond the typical assays to characterize the challenge stock, it is critical to have full genome sequencing and quantification of the particle:pfu ratio, as both can impact the virulence of the stock [21]. Periodic confirmation of virulence in animal models will be an important aspect of the long-term characterization of the isolate master and working stocks. Serial passage also impacts the EBOV genome, most notably by causing an extra uracil (U) residue to be inserted into a poly-U site of the gylcoprotein sequence, resulting in 8U variants instead of the wild-type 7U virus [22]. Challenge stocks consisting largely of an “8U” sequence resulted in a more protracted course of disease, prompting the authors of that study to recommend the use of challenge stocks consisting of predominantly “7U” challenge material moving forward [23]. The balance of 7U versus 8U affects processing of the glycoprotein for Ebola viruses, but does not appear to impact processing of the MARV glycoprotein.

Once the strain of the challenge agent has been selected, a particular isolate of that strain must be chosen for preparation and characterization. Isolate selection is based on several critical criteria:

1. Each is a low-passage stock derived from a clinical isolate. This is crucial to avoid genetic drift from selective pressure, which can impact a myriad of product development endpoints.
2. Each isolate is from a known fatal (human/clinical) case.
3. The whole genome RNA sequence is available from both passage 2 and 3.
4. Each isolate has been shown to cause systemic fatal disease in the 1000-pfu intramuscular nonhuman primate challenge model in natural history studies, demonstrating the utility of the model in the evaluation of filovirus countermeasures.
5. Each isolate has a well-documented lineage/pedigree which clearly defines the chain of custody and passage history, including but not limited to cell type used, multiplicity of infection or MOI, media utilized, and technique for harvesting and sequencing.

## 5. Selection of Viral Isolates

### 5.1. Zaire Ebolavirus

An EBOV virus used in several challenge studies conducted in 2014 to 2016 was isolated from the largest recorded outbreak in western Africa [24] and was isolated from a severe fatal disease. Prior to this, EBOV isolates of the Kikwit strain were heavily utilized in nonclinical testing, originating from a lethal human case in the 1995 EBOV outbreak that infected 315 patients, with a fatality rate of 81 percent [25]. The evaluation of EBOV vaccines using highly virulent strains of EBOV isolates ensured that the results provided a conservative measure of product efficacy. The use of a severe challenge isolate also provides assurance that a vaccine, which will potentially be administered to thousands of patients, will be an effective outbreak countermeasure. The case fatality rate of filovirus infections, and the realization that the first use of countermeasures may be in response to an outbreak when clinical efficacy has not yet been demonstrated, make a conservative evaluation of product efficacy critical in product development. This further highlights the rationale in selecting viral isolates, but EBOV isolates will not be described further since licensed vaccines and therapeutics are now available.

### 5.2. Marburg Marburgvirus

Known isolates of the marburgviruses can be separated into two major lineages (Marburg virus or MARV and Ravn virus or RAVV), but all virus isolates remain members of the same species Marburg marburgvirus. Genomic variation among the marburgviruses is greatest between these two lineages and estimated at 21.0–21.4 percent [26]. Early vaccine studies utilizing the glycoprotein from one lineage were able to elicit protection against the other lineage [27]; thus, a single MARV isolate may suffice for the construction of a vaccine to protect against currently known marburgviruses. A variety of different candidate vaccines have been assessed in the cynomolgus macaque model, including those with MARV glycoprotein expressed in a vesicular stomatitis virus [28], adenoviruses [27], modified vaccinia Ankara [29], encoded as a DNA vaccine [30], and protein-based vaccines assembled into virus-like particles [31] and viral replicons [32]. MARV isolates used in nonclinical efficacy studies to date have included MARV Angola, Musoke and Ci67, and RAVV [26].

Marburg virus *H. sapiens*-tc/ANG/2005/Angola-200501379 meets the key aspects for a MARV isolate suitable for use as a challenge material in nonclinical testing and evaluation under the Animal Rule. This MARV Angola strain is a human isolate at low passage from a fatal clinical case of an eight-month-old female from Uige, Angola, during the 2005 MARV outbreak [33]. The patient was hospitalized in March of 2005, and blood was collected during hospitalization from which the virus isolate was cultured. Passage history is documented, and the strain is accessible through special request to the Biodefense and Emerging Infections Research Resources Repository (BEI Resources). Nonclinical studies using the Angola strain of MARV have shown that infection is uniformly lethal, using a challenge dose of 1000 pfu via the intramuscular challenge route [34]. While these studies have utilized a small number of mock-vaccinated control animals, a recent natural history study assessing MARV infection in cynomolgus macaques demonstrated uniform lethality associated with fever and viremia, along with other key aspects of clinical MVD (unpublished data). U.S. government-supported natural history studies will be submitted to the FDA/Center for Biologics Evaluation and Research under a Type V Master File and will be available for reference from product sponsors.

### 5.3. Sudan Ebolavirus

SUDV represents a distinct species among ebolaviruses. SUDV was first identified as the cause of a hemorrhagic disease outbreak in 1976, comprising 284 cases, 53 percent of which were fatal [1]. Another large outbreak occurred in 2000, with 425 cases [5], which represented the largest outbreak of EVD on record until the 2014–2016 West Africa epidemic. The overall diversity among SUDV genomes is fairly low, with a maximum of 5.2 % variance in sequences collected from Sudan and Uganda [35]. Similarly to MARV, a number of candidate vaccines have been assessed in the cynomolgus macaque model, including those expressed by vesicular stomatitis virus [36], adenoviruses [29], protein-based vaccines assembled as virus-like particles [37], and viral replicons [38]. These studies utilized either Boniface or Gulu isolates of SUDV.

Sudan virus *H. sapiens*-tc/UG/2000/Gulu-200011676 meets the key aspects for a SUDV isolate suitable for use as a challenge material in nonclinical testing and evaluation under the Animal Rule. This SUDV Gulu strain is at low passage from a fatal clinical case. The isolate is from a 35-year-old male from Gulu, Uganda, during the 2000 outbreak. Blood containing the virus isolate was obtained shortly before the patient succumbed to illness. Passage history is documented, with only two passages, and the isolate may be obtained from BEI resources by special request similar to MARV Angola. Similar to MARV Angola, nonclinical vaccine efficacy studies utilizing SUDV Gulu as the challenge stock have been near uniformly lethal in small numbers of mock-vaccinated controls. Natural history studies have also commenced for SUDV Gulu, and to date, have confirmed near-uniform lethality at a 1000-pfu intramuscular challenge. Clinical disease manifestations have been similar to those observed in humans (unpublished data). This study report will also be submitted to the FDA/Center for Biologics Evaluation and Research for cross-reference by sponsors under a Type V Master File.

## 6. Path Forward and Conclusions

The development of products under the FDA Animal Rule requires well-characterized and understood animal models with well-defined challenge virus isolates. In order to be consistent with the requirements of the FDA Animal Rule, the challenge agent should be a low-passage isolate from a human fatal case that produces an infection in the animal model, that can be used to evaluate medical countermeasures intended to treat the authentic human disease. The isolates for MARV and SUDV are most consistent with these requirements. Given the likelihood that vaccines against MARV and SUDV will proceed towards licensure via the FDA Animal Rule, we recommend at this time that the primary focus, in terms of nonclinical efficacy studies, should be on the Angola and Gulu isolates for MARV and SUDV noted above. The adoption of these challenge isolates will allow the standardization of vaccine development across programs and will simplify the interpretation of nonclinical efficacy studies for both funding and regulatory authorities. Barring any large outbreaks of MARV or SUDV in the near term, the MARV Angola and SUDV Gulu isolates represent recent virus isolates that have well-documented clinical and laboratory histories, meet the guidelines of the FDA Animal Rule, and have demonstrated clinical disease endpoints in nonclinical studies that are consistent with human disease. The Biomedical Advanced Research and Development Authority (BARDA), in coordination with the NIH and DOD, is supporting natural history studies of MARV Angola and SUDV Gulu. Product sponsors will be able to cross-reference Type V Master Files for these natural history studies under their respective regulatory submissions.

We have concentrated on describing the identification of the appropriate SUDV and MARV isolates for product evaluation under the FDA Animal Rule in this manuscript because prophylactic and therapeutic countermeasures against these viruses is a BARDA priority. We believe the same considerations used to select the SUDV and MARV isolates will be applicable if additional filovirus strains such as the Bundibugyo or Tai Forest viruses become BARDA priorities for medical countermeasure development or if new and novel Ebola virus strains arise from environmental exposures and become a medical priority.

The zoonotic nature of filoviruses and the sporadic nature of outbreaks suggests that new and equally lethal versions may emerge. Thus, it may be appropriate to obtain samples from new or previous outbreaks for testing to ensure the effectiveness of medical countermeasures against different isolates. In that light, continued biosurveillance will be a key aspect of ongoing filovirus research, which has recently uncovered a potential sixth species of ebola virus, *Bombali ebolavirus* [39]. It will be important to evaluate the degree of cross-protection that may be conferred across virus isolates. For example, nonclinical efficacy studies may focus on a specific challenge stock in the product development pathway for a MARV vaccine. However, if and when new isolates emerge with diversity, specifically in the glycoprotein gene, assessments of cross-reactivity of the immune response through in vitro neutralization assays will provide key data on whether or not protection against new isolates will be likely.

## Data Availability

Not applicable.

## References

[1] Kuhn J.H., Adachi T., Adhikari N.K., Arribas J.R., Bah I.E., Bausch D.G., Bhadelia N., Borchert M., Brantsæter A.B., Brett-Major D.M. (2019). New filovirus disease classification and no-menclature. Nat. Rev. Microbiol..

[2] Rojas M., Monsalve D.M., Pacheco Y., Acosta-Ampudia Y., Ramírez-Santana C., Ansari A.A., Gershwin M.E., Anaya J.-M. (2019). Ebola virus disease: An emerging and re-emerging viral threat. J. Autoimmun..

[3] Henao-Restrepo A.M., Camacho A., Longini I.M., Watson C.H., Edmunds W.J., Egger M., Carroll M.W., Dean N.E., Diatta I., Doumbia M. (2017). Efficacy and effectiveness of an rVSV-vectored vaccine in preventing Ebola virus disease: Final results from the Guinea ring vaccination, open-label, cluster-randomised trial (Ebola Ça Suffit!). Lancet.

[4] World Health Organization (2019). Preliminary Results on the Efficacy of rVSV-ZEBOV-GP Ebola Vaccine Using the Ring Vaccination Strategy in the Control of an Ebola Outbreak in the Democratic Republic of the Congo: An Example of Integration of Research into Epidemic Response.

[5] Okware S.I., Omaswa F.G., Zaramba S., Opio A., Lutwama J.J., Kamugisha J., Rwaguma E.B., Kagwa P., Lamunu M. (2002). An outbreak of Ebola in Uganda. Trop. Med. Int. Health.

[6] Sanchez A., Lukwiya M., Bausch D., Mahanty S., Sanchez A.J., Wagoner K.D., Rollin P. (2004). Analysis of Human Peripheral Blood Samples from Fatal and Nonfatal Cases of Ebola (Sudan) Hemorrhagic Fever: Cellular Responses, Virus Load, and Nitric Oxide Levels. J. Virol..

[7] MacNeil A., Farnon E.C., Wamala J.F., Okware S.I., Cannon D.L., Reed Z., Towner J.S., Tappero J.W., Lutwama J.J., Downing R. (2010). Proportion of Deaths and Clinical Features in Bundibugyo Ebola Virus Infection, Uganda. Emerg. Infect. Dis..

[8] Centers for Disease Control and Prevention (2014). Outbreaks Chronology: Marburg Hemorrhagic Fever. https://www.cdc.gov/vhf/marburg/outbreaks/chronology.html.

[9] Glaze E.R., Roy M.J., Dalrymple L.W., Lanning L.L. (2015). A Comparison of the Pathogenesis of Marburg Virus Disease in Humans and Nonhuman Primates and Evaluation of the Suitability of These Animal Models for Predicting Clinical Efficacy under the ‘Animal Rule’. Comp. Med..

[10] U.S. Food and Drug Administration (2015). Product Development under the Animal Rule.

[11] Wolf J., Jannat R., Dubey S., Troth S., Onorato M., Coller B.-A., Hanson M., Simon J. (2021). Development of Pandemic Vaccines: ERVEBO Case Study. Vaccines.

[12] Ledgerwood J., Costner P., Desai N., Holman L., Enama M., Yamshchikov G., Mulangu S., Hu Z., Andrews C., Sheets R. (2010). A replication defective recombinant Ad5 vaccine expressing Ebola virus GP is safe and immunogenic in healthy adults. Vaccine.

[13] Martin J.E., Sullivan N.J., Enama M.E., Gordon I.J., Roederer M., Koup R.A., Bailer R.T., Chakrabarti B.K., Bailey M.A., Gomez P.L. (2006). A DNA Vaccine for Ebola Virus Is Safe and Immunogenic in a Phase I Clinical Trial. Clin. Vaccine Immunol..

[14] Wolfe D.N., Zarrabian A.G., Disbrow G.L., Espeland E.M. (2019). Progress towards a vaccine against Ebola to meet emergency medical countermeasure needs. Vaccine.

[15] Dulin N., Spanier A., Merino K., Hutter J.N., Waterman P.E., Lee C., Hamer M.J. (2020). Systematic review of Marburg virus vaccine nonhuman primate studies and human clinical trials. Vaccine.

[16] Wolfe D.N., Taylor M.J., Zarrabian A.G. (2020). Lessons learned from *Zaire ebolavirus* to help address urgent needs for vaccines against *Sudan ebolavirus* and Marburg virus. Hum. Vaccines Immunother..

[17] Roozendaal R., Hendriks J., van Effelterre T., Spiessens B., Dekking L., Solforosi L., Czapska-Casey D., Bockstal V., Stoop J., Splinter D. (2020). Nonhuman primate to human immunobridging to infer the protective effect of an Ebola virus vaccine candidate. NPJ Vaccines.

[18] Marzi A., Robertson S.J., Haddock E., Feldmann F., Hanley P.W., Scott D.P., Stron J.E., Kobinger G., Best S.M., Feldmann H. (2015). VSV-EBOV rapidly protects macaques against infection with the 2014/15 Ebola virus outbreak strain. Science.

[19] Park D.J., Dudas G., Wohl S., Goba A., Whitmer S.L.M., Andersen K.G., Sealfon R.S., Ladner J.T., Kugelman J.R., Matranga C.B. (2015). Ebola Virus Epidemiology, Transmission, and Evolution during Seven Months in Sierra Leone. Cell.

[20] Ruedas J.B., Arnold C.E., Palacios G., Connor J.H. (2018). Growth-Adaptive Mutations in the Ebola Virus Makona Glycoprotein Alter Different Steps in the Virus Entry Pathway. J. Virol..

[21] Alfson K.J., Avena L.E., Beadles M.W., Staples H., Nunneley J.W., Ticer A., Dick E.J., Owston M.A., Reed C., Patterson J.L. (2015). Particle-to-PFU Ratio of Ebola Virus Influences Disease Course and Survival in Cynomolgus Macaques. J. Virol..

[22] Kugelman J.R., Lee M.S., Rossi C.A., McCarthy S.E., Radoshitzky S., Dye J.M., Hensley L., Honko A., Kuhn J.H., Jahrling P.B. (2012). Ebola Virus Genome Plasticity as a Marker of Its Passaging History: A Comparison of In Vitro Passaging to Non-Human Primate Infection. PLoS ONE.

[23] Trefrey J.C., Wollen S.E., Nasar F., Shamblin J.D., Kern S.J., Bearss J.J., Jefferson M.A., Chance T.B., Kugelman J.R., Ladner J.T. (2015). Ebola Virus Infections in Nonhuman Primates Are Temporally Influenced by Glycoprotein Poly-U Editing Site Populations in the Exposure Material. Viruses.

[24] Baize S., Pannetier D., Oestereich L., Rieger T., Koivogui L., Magassouba N., Soropogui B., Sow M.S., Keïta S., De Clerck H. (2014). Emergence of Zaire Ebola Virus Disease in Guinea. N. Engl. J. Med..

[25] Muyembe-Tamfum J.J., Kipasa M., Kiyungu C., Colebunders R. (1999). Ebola Outbreak in Kikwit, Democratic Republic of the Congo: Discovery and Control Measures. J. Infect. Dis..

[26] Peterson A.T., Holder M.T. (2012). Phylogenetic assessment of filoviruses: How many lineages of Marburg virus?. Ecol. Evol..

[27] Swenson D.L., Warfield K.L., Larsen T., Alves D.A., Coberley S.S., Bavari S. (2008). Monovalent virus-like particle vaccine protects guinea pigs and nonhuman primates against infection with multiple Marburg viruses. Expert Rev. Vaccines.

[28] Mire C.E., Geisbert J.B., Agans K.N., Satterfield B., Versteeg K.M., Fritz E.A., Feldmann H., Hensley L., Geisbert T.W. (2014). Durability of a Vesicular Stomatitis Virus-Based Marburg Virus Vaccine in Nonhuman Primates. PLoS ONE.

[29] Callendret B., Vellinga J., Wunderlich K., Rodriguez A., Steigerwald R., Dirmeier U., Cheminay C., Volkmann A., Brasel T., Carrion R. (2018). A prophylactic multivalent vaccine against different filovirus species is immunogenic and provides protection from lethal infections with Ebolavirus and Marburgvirus species in non-human primates. PLoS ONE.

[30] Grant-Klein R.J., Altamura L.A., Badger C.V., Bounds C.E., Van Deusen N.M., Kwilas S.A., Vu H.A., Warfield K.L., Hooper J., Hannaman D. (2015). Codon-Optimized filovirus DNA vaccines delivered by intramuscular electroporation protect cynomolgus macaques from lethal Ebola and Marburg virus challenges. Hum. Vaccines Immunother..

[31] Dye J.M., Warfield K.L., Wells J.B., Unfer R.C., Shulenin S., Vu H., Nichols D.K., Aman M.J., Bavari S. (2016). Virus-Like Particle Vaccination Protects Nonhuman Primates from Lethal Aerosol Exposure with Marburgvirus (VLP Vaccination Protects Macaques against Aerosol Challenges). Viruses.

[32] Hevey M., Negley D., Pushko P., Smith J., Schmaljohn A. (1998). Marburg virus vaccines based upon alphavirus replicons protect guinea pigs and nonhuman primates. Virology.

[33] Towner J.S., Khristova M.L., Sealy T.K., Vincent M.J., Erickson B.R., Bawiec D.A., Hartman A., Comer J.A., Zaki S.R., Ströher U. (2006). Marburgvirus Genomics and Association with a Large Hemorrhagic Fever Outbreak in Angola. J. Virol..

[34] Blair P.W., Keshtkar-Jahromi M., Psoter K.J., Reisler R.B., Warren T.K., Johnston S.C., Goff A.J., Downey L.G., Bavari S., Cardile A.P. (2018). Virulence of Marburg Virus Angola Compared to Mt. Elgon (Musoke) in Macaques: A Pooled Survival Analysis. Viruses.

[35] Carroll S.A., Towner J.S., Sealy T.K., McMullan L.K., Khristova M.L., Burt F.L., Swanepoel R., Rollin P.E., Nichol S.T. (2013). Mo-lecular Evolution of Viruses of the Family *Filoviridae* Based on 97 Whole-Genome Sequences. J. Virol..

[36] Geisbert T.W., Geisbert J.B., Leung A., Daddario-DiCaprio K.M., Hensley L.E., Grolla A., Feldmann H. (2009). Single-Injection Vaccine Protects Nonhuman Primates against Infection with Marburg Virus and Three Species of Ebola Virus. J. Virol..

[37] Warfield K.L., Dye J.M., Wells J.B., Unfer R.C., Holtsberg F.W., Shulenin S., Vu H., Swenson D.L., Bavari S., Aman M.J. (2015). Homologous and Heterologous Protection of Nonhuman Primates by Ebola and Sudan Virus-Like Particles. PLoS ONE.

[38] Herbert A.S., Kuehne A.I., Barth J.F., Ortiz R.A., Nichols D.K., Zak S.E., Stonier S.W., Muhammad M.A., Bakken R.R., Prugar L.I. (2013). Venezuelan Equine Encephalitis Virus Replicon Particle Vaccine Protects Nonhuman Primates from Intramuscular and Aerosol Challenge with Ebolavirus. J. Virol..

[39] Goldstein T., Anthony S.J., Gbakima A., Bird B.H., Bangura J., Tremeau-Bravard A., Belaganahalli M.N., Wells H., Dhanota J.K., Liang E. (2018). Discovery of a new ebolavirus (Bombali virus) in molossid bats in Sierra Leone. Nat. Microbiol..

